# Supplementary material for: Directed closure coefficient and its patterns
Source: PLoS One. 2021 Jun 25;16(6):e0253822. doi: 10.1371/journal.pone.0253822 (PMC8232453; doi:10.1371/journal.pone.0253822)
Supplement: Attachment — Submitted filename: review.pdf [file pone.0253822.s001.pdf]

Title: Directed closure coefficient and its patterns
Authors: Mingshan Jia, Bogdan Gabrys and Katarzyna Musial

Verdict: Minor revision

Summary

In this paper the authors study the formation of triangles in directed networks. They do so, using the recently developed notion of the closure coefficient. While the local clustering coefficient measures the probability that two neighbors of a node are also neighbors, the closure coefficient measures the probability that a neighbor of your neighbor is also your neighbor. In other words, the probability of the given node to close a triangle.
The authors first introduce the closure coefficients in the setting of directed graph. This yields eight different coefficients. They then categorize these into four categories, which are meant to represent different closure patterns. They also provide extensions to weighted and signed directed networks. They then use the four categories to investigate the importance of closure coefficients for characterizing the network structure. For this the authors look at 24 directed networks in 6 different domains. The concept of closure coefficients is then applied for classifying the domain of the network and for link prediction. In particular, the authors compare the impact of the closure coefficient to the success these task to that of the classic clustering coefficients. They conclude that for a wide range of networks, the closure coefficients improve the success rate, implying that triangle formation in directed networks via closure is more descriptive than captured via the classical clustering coefficient.

Review

The study of triangles in complex networks is fast and relevant. This is particularly true for directed networks, given the multiple types of triangles one can observe. This work provides a nice addition to this endeavor and provides some new insights into the differences between triangular structures and their impact on relevant activities on networks. I thus think that this work is worthy of publication.
Overall, I found the manuscript to be well written and easy to read. The structure is clear and all necessary details are presented. One place were some improvements could be made (especially with respect to reproducibility) is in the section on classification. Here the authors use several methods for classification. However, it is not clear if these different methods are included in the code package they refer to. I was unable to deduce this from Python code files on the GitHub page. I would recommend the authors to either explicitly mention that everything needed for reproducing their experiments is in the provided code. Or include some additional information (potentially in supplementary) so that other researchers can easily repeat the same experiments.
There is also some problem with the framing of this work. Contrary to what the abstract and exposition seem to suggest, it is not the first to extend the concept of closure coefficients to directed networks. Reference [60] already did this. The authors comment on this in Section 5, claiming that they give a more general definition. However, upon inspecting [60] I found the definitions for directed networks to be the same. What is new in this manuscript is the classification of the four different categories of triangle closures as well as the extension to

weighted and signed graphs. In light of this, I would strongly recommend the authors to frame their work more accurately in the existing literature. Especially, considering [60].
In summary, while I definitely think this is interesting work that deserves publication, the current manuscript still needs to be improved a bit. The most important part is the positioning with respect to the literature. I would recommend the authors to make crystal clear what the new and novel additions of this work are. Below I include some other small comments and suggestions.

Comments/Suggestions

- Page 1, Abstract, line -5: "...we propose four closure patterns."
- Page 9, lines 283 and 284: There seems to be an additional space between the comma and the remaining numbers in 3,783 and 24,186.
- Page 9, Lines 285-286: I guess that ρ refers to Pearsons correlations coefficient. If so then it is important to check that the degree distribution of the network resembles a distribution with finite variance. Because if this is not the cause than Pearson becomes size dependent and will converge to zero as the size of the network increases. Independently on what the correlation between degrees and closure coefficient.
- Section 3.4: The exposition here is not entirely accurate. Computational complexity is always a worst-case scenario. So the complexity should be $O(k_{max})$ (maximum degree) instead of $O(\bar{k})$. What I think this section is meant to convey is that in practice the algorithm works very fast for large networks. If this is the case, then I would simply state it as that and not use computation complexity analysis.
- Page 11, lines 342-348: Are closure coefficients expected to always converge to an informative number in relevant network models? This is extremely relevant if we are comparing the magnitude of the numbers.

  For example, we know that the global clustering coefficient always converges to zero for degree distributions with infinite variance, independent on the triangle structure.
- Page 11, lines 349-352: Some scatter plots seem to show absolute chaos. Especially those for web graphs. So I am a bit skeptical about the general conclusion that is made here.
- Page 11, lines 352-355: I am Not completely sure about this claim. Pearson measures for possible linear dependencies between quantities. So not too strong values might also indicate more complex non-linear relationships. Maybe including different measures for correlations would help strengthen the claim.
- Page 13, Table 2: Is there any explanation why the Random Forest classification would work less well with closure coefficients?
- Page 13, lines 404-406: This seems somewhat trivial to me. Adding more characteristics would naturally improve any classification right?
