# Supplementary material for: Directed closure coefficient and its patterns
Source: PLoS One. 2021 Jun 25;16(6):e0253822. doi: 10.1371/journal.pone.0253822 (PMC8232453; doi:10.1371/journal.pone.0253822)
Supplement: Attachment — Submitted filename: Response to Reviewers.pdf [file pone.0253822.s002.pdf]

PONE-D-21-06621
Directed closure coefficient and its patterns
PLOS ONE

Rebuttal Letter

Dear Editor,

Thank you very much for handling this submission. According to the reviewers' comments and suggestions, we have carefully revised the manuscript.

The main changes include: (i) We have made publicly available the codes of all the experiments through `https://github.com/MingshanJia/explore-local-structure`; (ii) We have broadened our literature review and included two more articles discussing the relationship between the community structure and the clustering coefficient; (iii) We have rewritten the section of related work and highlighted the novelty of our work; (iv) We have rerun the experiment of network classification and discussed the results more thoroughly with the support of newly added confusion matrices. Enclosed please find point-to-point responses to the reviewing reports.

Kind regards,
Mingshan Jia, Bogdan Gabrys and Katarzyna Musial

Reviewer 1

Reviewer Point P 1.1 — It is not clear if these different methods are included in the code package they refer to. I was unable to deduce this from Python code files on the GitHub page. I would recommend the authors to either explicitly mention that everything needed for reproducing their experiments is in the provided code. Or include some additional information (potentially in supplementary) so that other researchers can easily repeat the same experiments.

Authors response: We have further included the codes of all the experiments in this paper. We also provided a README file in the shared repository, so other research can easily understand and repeat our experiments.

Reviewer Point P 1.2 — I would strongly recommend the authors to frame their work more accurately in the existing literature. Especially, considering [60]. The most important part is the positioning with respect to the literature. I would recommend the authors to make crystal clear what the new and novel additions of this work are.

Authors response: To address this point, we have rewritten the section of related work and highlighted the novelty of our work. We have also changed the order of the referenced works and clearly stated how our work is different from reference [1].

Other comments/suggestions

Reviewer Comment C 1.1 — Page 1, Abstract, line -5: "...we propose four closure patterns."

Authors action: We have revised the sentence as suggested.

Reviewer Comment C 1.2 — Page 9, lines 283 and 284: There seems to be an additional space between the comma and the remaining numbers in 3,783 and 24,186.

Authors response: We have double checked the manuscript and there is no additional space between the comma and the numbers. The seemingly wider space is caused by auto formatting.

Reviewer Comment C 1.3 — Reviewer 1's third comment. Page 9, Lines 285-286: I guess that ρ refers to Pearsons correlations coefficient. If so then it is important to check that the degree distribution of the network resembles a distribution with finite variance. Because if this is not the cause than Pearson becomes size dependent and will converge to zero as the size of the network increases. Independently on what the correlation between degrees and closure coefficient.

Authors response: Yes, ρ refers to the Pearson correlation coefficient. Our case study is conducted on a real-world network (TR-BTCAlpha) with a finite number of nodes (3,783) and a finite number of edges (24,186). Therefore, the variance of node degree is also finite. In Figure 1, we show the node degree distribution, the node strength distribution and denote their variances for the studied network.

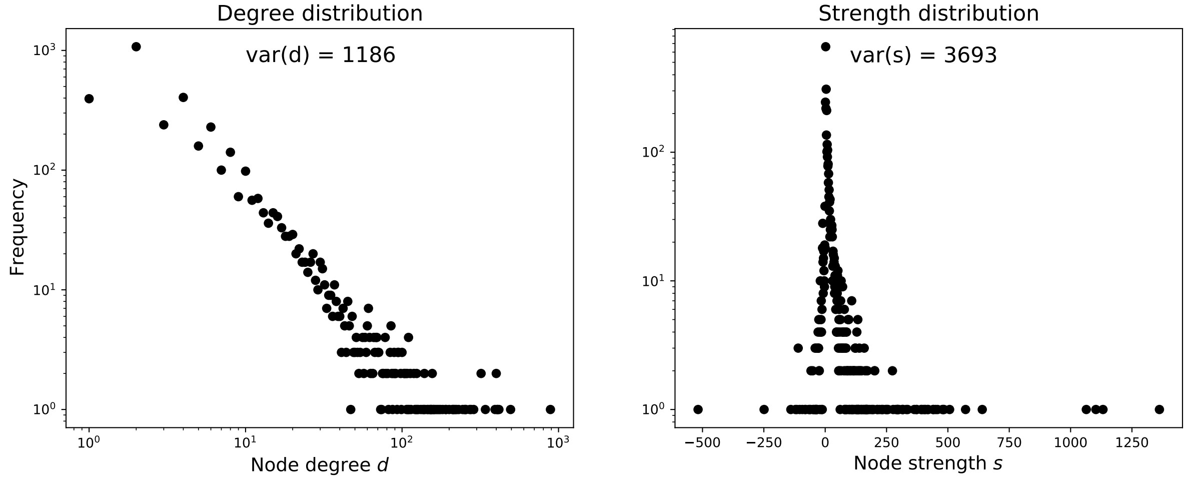

Figure 1: Node degree distribution and node strength distribution of network TR-BTCAlpha.

Authors action: In the revised manuscript, we clearly point out that ρ is the Pearson correlation coefficient.

Reviewer Comment C 1.4 — Section 3.4: The exposition here is not entirely accurate. Computational complexity is always a worst-case scenario. So the complexity should be $O(k_{max})$ (maximum degree) instead of $O(\bar{k})$. What I think this section is meant to convey is that in practice the

algorithm works very fast for large networks. If this is the case, then I would simply state it as that and not use computation complexity analysis.

Authors response: We didn't mention explicitly here that $O(\bar{k})$ is the average-case complexity. The complexity analysis also studies the average-case complexity because the worst cases may be very rare in practice (e.g. nodes with maximum degree are very rare in many real-world networks), and the average-case complexity may be more accurate measure of an algorithm's performance.

Authors action: In the revised manuscript, we explain explicitly that $O(\bar{k})$ and is the average-case complexity for computing the local directed closure coefficient, and $O(|V| \cdot \bar{k}^2)$ is the average-case complexity for computing the average directed closure coefficient.

Reviewer Comment C 1.5 — Page 11, lines 342-348: Are closure coefficients expected to always converge to an informative number in relevant network models? This is extremely relevant if we are comparing the magnitude of the numbers. For example, we know that the global clustering coefficient always converges to zero for degree distributions with infinite variance, independent on the triangle structure.

Authors response: Our analysis is conducted on 24 real world networks from six domains, not on network models. As the number of nodes and the number of edges of each network are fixed and finite, the average directed clustering coefficient ($\overline{C^{\mathcal{D}}}$) and the average directed closure coefficient ($\overline{E^{\mathcal{D}}}$) we reported in Table 1 are determined by these networks and their respective structures. The study of the convergence of the closure coefficients using different network models and investigations of whether they converge to an "informative number" would indeed be interesting though due its nature and scale it is outside of the scope of our paper. Thank you for the suggestion which we will investigate as part of our future research.

Reviewer Comment C 1.6 — Page 11, lines 349-352: Some scatter plots seem to show absolute chaos. Especially those for web graphs. So I am a bit skeptical about the general conclusion that is made here.

Authors response: Visual inspection of Figure 7 indicates that the plots within one type of network (in the same row) are more similar than the plots in between these types of networks (between rows). This observation is also the motivation of our following network classification task. WEB-BERKSTAN and WEB-GOOGLE contains a large number of nodes (685K and 876K respectively). The "chaos" in these two networks is inevitable due to the nature of scatter plot and the limited paper space, but in our opinion, the overall conclusion is supported by the plots and we have therefore retained it in the paper.

Authors action: We have added more discussion supporting our observations and the drawn conclusion in the revised manuscript.

Reviewer Comment C 1.7 — Page 11, lines 352-355: I am Not completely sure about this claim. Pearson measures for possible linear dependencies between quantities. So not too strong values might also indicate more complex non-linear relationships. Maybe including different measures for correlations would help strengthen the claim.

Authors response: We agree with the reviewer that a pair of variables with low Pearson correlation coefficient might have some complex non-linear relationship between them. Other commonly used correlation coefficients include the Spearman rank correlation coefficient and the Kendall rank correlation coefficient. Both coefficients measure the ordinal association between two variables (see if there is a monotonic relationship between the two quantities). In Table 1 we report all three correlation coefficients between the directed closure coefficient and the directed clustering coefficient.

Comparing Spearman's ρ with Pearson's r, we observe that in most networks ρ is higher than r (or lower when the coefficients are negative). This is naturally expected because a rank correlation is less strict than a linear correlation (the two variables don't need to change at the same rate). We also find that all four trust networks, one web graph (WEB-NOTREDAME) and two word adjacency networks (WA-JAPANESE and WA-FRENCH) exhibit very strong Spearman's correlation. However, the correlation is not that strong in the remaining 18 networks. It indicates that we cannot describe the relationship between the two variables by simply using these correlation coefficients. The Pearson's correlation coefficient reported in Figure 7 is a simple and first analysis to demonstrate that the information contained in the proposed directed closure coefficient is different from that contained in the classic directed clustering coefficient. The following experiments continue to show how the complementary information can be used in other network analysis tasks including network classification and link prediction.

Reviewer Comment C 1.8 — Page 13, Table 2: Is there any explanation why the Random Forest classification would work less well with closure coefficients?

Authors response:

(a) Avg. error matrix of RF with clustering patterns

Act. \ Pred.	TR	FW	Cit	SW	Web	WA
TR	**2.96**	0	0	0	0	1.04
FW	0	**2**	0	1	1	0
Cit	0.01	0.01	**2.99**	0.99	0	0
SW	0	0	0	**4**	0	0
Web	1	0	0	0	**3**	0
WA	1.34	0	0	0	0	**2.66**

(b) Avg. error matrix of RF with closure patterns

Act. \ Pred.	TR	FW	Cit	SW	Web	WA
TR	**3**	0	1	0	0	0
FW	0	**2.86**	0.98	0	0.16	0
Cit	0	0	**3**	1	0	0
SW	0	0	1	**2.35**	0	0.65
Web	1	1	0	0	**2**	0
WA	0	0	0	0.03	0	**3.97**

Figure 2: Average confusion matrices of Random Forest model with different feature sets.

To answer this question, we further compare the confusion matrix of Random Forest classifier using clustering patterns with the confusion matrix of Random Forest using closure patterns (Figure 2). We can see that Random Forest model with closure patterns features is not very good at classifying software networks and web graphs: the accuracies are 0.588 and 0.5, respectively. In contrast, when clustering patterns are used as features in Random Forest, the model can perfectly classify software networks, and the accuracy of classifying web graphs is 0.75. In short, the underlying reason is that Random Forest is not as efficient at using closure patterns in categorising software networks and web graphs.

Although the overall accuracy of Random Forest with closure patterns is a little bit lower than that of Random Forest with clustering patterns, we can also observe in Figure 2 that Random Forest with closure

Table 1: Three correlation coefficients between the directed clustering coefficient and the directed closure coefficient.

Network	Pearson's r	Kendall's τ	Spearman's ρ
Tr-BTCAlpha	0.199	0.666	0.865
Tr-BTC-OTC	0.213	0.674	0.871
Tr-Advogato	0.419	0.675	0.857
Tr-Epinions	0.457	0.838	0.961
FW-Mangrove	-0.212	-0.107	-0.157
FW-BayWet	0.106	0.106	0.167
FW-BayDry	0.129	0.123	0.196
FW-LittleRock	0.518	0.465	0.647
Cit-Cora	0.435	0.538	0.715
Cit-HepTh	0.380	0.300	0.425
Cit-HepPh	0.249	0.229	0.329
Cit-Citeseer	0.437	0.613	0.795
SW-Weka	0.097	0.408	0.573
SW-Lucene	0.012	0.328	0.475
SW-JUNG	-0.296	-0.557	-0.707
SW-JDK	-0.294	-0.529	-0.679
Web-Stanford	0.195	0.283	0.405
Web-NotreDame	0.465	0.800	0.942
Web-BerkStan	0.209	0.319	0.454
Web-Google	0.395	0.527	0.704
WA-Japanese	0.029	0.659	0.868
WA-English	-0.076	0.280	0.472
WA-French	0.050	0.679	0.878
WA-Spanish	-0.021	0.500	0.724

patterns is better at classifying food webs and word adjacency networks, and slightly better at classifying trust networks. It indicates that the information contained in the closure patterns are complementary to the information contained in the clustering patterns. Therefore, as expected, combining both the clustering patterns and the closure patterns yields the best classification accuracy.

Authors action: We have included the average confusion matrices and the analysis in the revised manuscript.

Reviewer Comment C 1.9 — Page 13, lines 404-406: This seems somewhat trivial to me. Adding more characteristics would naturally improve any classification right?

Authors response: Adding more features may lead to improvement in classification, but only when the new features are both informative and complementary in the type of information they carry to the existing features as it has been observed by adding the closure coefficients to the clustering coefficients in the extended feature set. To support this claim, we conduct the following statistical test on Gradient Boosted Decision Tree model.

First we state two null hypotheses:

Null Hypothesis 1: Adding four random features to clustering patterns will improve the accuracy of classification, compared to only using the clustering patterns.

Null Hypothesis 2: Adding four random features to clustering patterns will achieve at least the accuracy of adding the closure patterns to the clustering patterns.

Statistical test setup. We generate random features from a uniform distribution over 0 to 1. Four columns of random features are appended to the four clustering patterns, and put into the Gradient Boosted Decision Tree model. As previously, we use leave-one-out cross validation to evaluate the classification performance.

Statistical test result. In Figure 3, we report the accuracy distribution of $1,000$ different sets of random features. To compare, we calculate the accuracy of using two baseline feature sets: one set including only the clustering patterns (accuracy is 0.667) , and another set including both the clustering patterns and the closure patterns (accuracy is 0.792). We finally have p-value of Null Hypothesis 1 equals to 0.052 (52 out of $1,000$ sets of random features achieve better accuracies). It is slightly over the default threshold 0.05. Thus, Null Hypothesis 1 is retained: adding random features to clustering patterns may improve the accuracy of classification.

However, p-value of Null Hypothesis 2 equals to 0.001 (only 1 out 1000 sets of random features achieves the same accuracy as adding closure patterns). Thus, Null Hypothesis 2 is confidently rejected, and the statistical significance of the improvement brought by adding closure patterns is demonstrated.

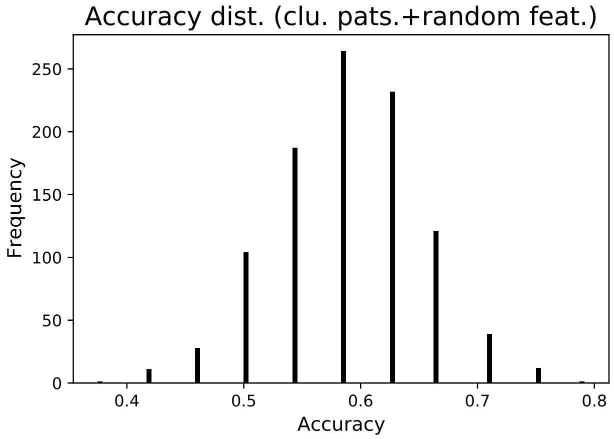

Figure 3: Accuracy distribution of GBDT with clustering patterns and random features.

Reviewer 2

Reviewer Point P 2.1 — First of all, the authors can better highlight the paper's interest by broadening the literature review to show the importance of the issue. I suggest incorporating and commenting on literature dealing with the clustering coefficient related to the community structure.

Authors response: As suggested, we have broadened our literature review by including the two suggested articles [2, 3] which are indeed relevant to our discussions. Ji et al. [2] proposed a novel divisive algorithm for community detection using the local clustering coefficient. Orman et al. [3]

studied and revealed the relationship between the community strucuture and the clustering coefficient of complex networks.

Reviewer Point P 2.2 — Second, make sure that the code and the data are available to reproduce and diffuse their work more efficiently.

Authors response: Similarly to our reply to Reviewer 1, we have further included the codes of all the experiments in this paper in the associated GITHUB repository. We also provided a README file in the shared repository, so other research can easily understand and repeat our experiments.

References

[1] Yin H, Benson AR, Ugander J. Measuring directed triadic closure with closure coefficients. Network Science. 2020;8(4):551–573.

[2] Ji Q, Li D, Jin Z. Divisive Algorithm Based on Node Clustering Coefficient for Community Detection. IEEE Access. 2020;8:142337–142347.

[3] Orman K, Labatut V, Cherifi H. An empirical study of the relation between community structure and transitivity. In: Complex Networks. Springer; 2013. p. 99–110.
